# Can site performance be predicted? Results of an evaluation of the performance of a site selection questionnaire in five multicentre trials

**DOI:** 10.1186/1745-6215-16-S2-P176

**Published:** 2015-11-16

**Authors:** Dawn Coleby, Diane Whitham, Lelia Duley

**Affiliations:** 1 Nottingham Clinical Trials Unit, University of Nottingham, Nottingham, UK

## Background

Site Selection Questionnaires (SSQs) have evolved to become “best” practice for commercial and non-commercial clinical trials. There has been limited evaluation of such questionnaires, however, and there is no generally accepted tool or methodology for selecting suitable sites for inclusion in a trial. Nottingham Clinical Trials Unit introduced a site selection questionnaire in 2010, this paper will present evaluation of the questionnaire’s performance in five trials.

## Method

Sites expressing an interest in participating in one of the 5 trials were sent an SSQ for completion by the PI; responses were made available to the CI to use if they wished. Once recruitment started, questionnaire responses were assessed ‘blind’ to site and PI identifiers, using predefined criteria: not returned, pool of participants, staff resources, trials experience, competing trials, and equipoise.

## Results

Overall in these 5 trials 421 questionnaires were sent out, and 155 sites selected. Results of the blinded assessment and agreement between this assessment and the CI will be presented and discussed. Site performance based on time to recruitment of first participant, and ratio of actual to target recruitment, will be compared for sites included and excluded based on the blind assessment.

## Discussion

Site selection is a key element for quality and efficiency in clinical trials. Results from this evaluation contribute to the understanding of how a well developed Site Selection Questionnaire can improve selection of sites in large multicentre trials.

